# Color Doppler findings of post-biopsy arteriovenous fistula in renal transplant

**DOI:** 10.4103/0971-4065.43696

**Published:** 2008-07

**Authors:** F. Shaheen, A. Hakeem, M. Singh, T. Gojwari, H. Shafi, M. Wani, S. Rasool

**Affiliations:** Department of Radio-Diagnosis, Medicine and Urology, SK Institute of Medical Sciences, Jammu and Kashmir - 190 011, India

**Keywords:** Arteriovenous fistula, color Doppler, post-biopsy

## Abstract

Post biopsy arterio-venous fistula in renal transplant range in incidence from 15-16%. Spontaneous resolution of 75% A-V fistulas is seen within four weeks. We report a patient with post biopsy arterio-venous fistula who had developed unexplained hypertension with no definite feature of rejection on biopsy. Doppler application revealed an arterio-venous fistula which showed spontaneous resolution in six weeks.

## Introduction

In renal transplants post biopsy fistulas are well known. Most resolve spontaneously only few which are large need intervention. We report a case in whom we had done renal biopsy and who developed post biopsy AV fistula which we documented on color Doppler. He was followed for 6 weeks after which we noticed a spontaneous resolution. Most of the post biopsy AV fistula need follow up and are likely to resolve after some period.

## Case Report

**Fig. 1:**
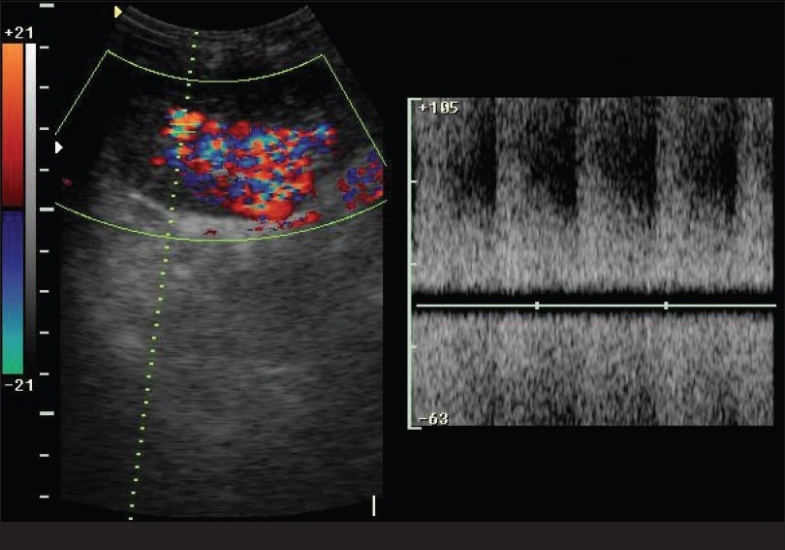
Showing turbulent flow on pulse wave doppler study

**Fig. 2:**
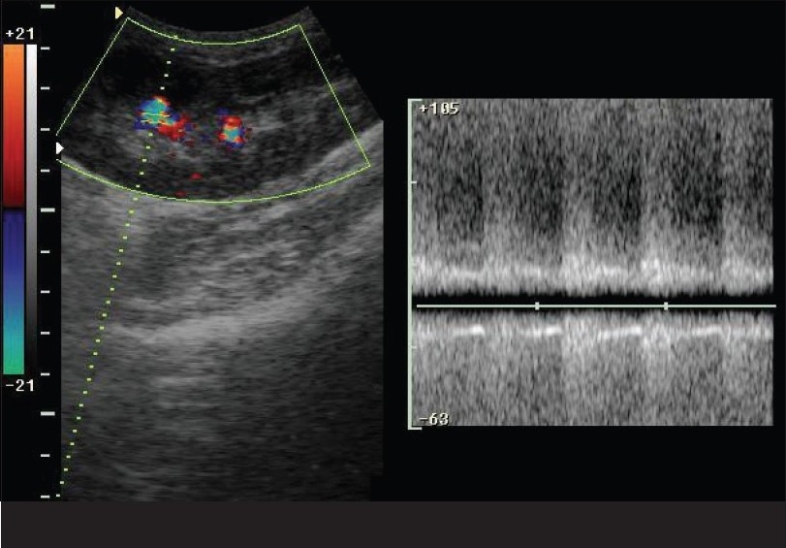
Arterializations of the venous waveform along with peri-vascular random color assignment on pulse wave Doppler flow

We evaluated a 46-year-old male, who had received live donor renal transplant one year ago, for a recent onset of hypertension. We performed three renal biopsies over a period of three months with no evidence of chronic transplant rejection. The patient was referred to the Radiology department for ultrasonography (USG) Doppler of the transplant. We performed a color Doppler study of the transplant kidney, which revealed an arteriovenous (AV) fistula in the lower pole involving lobar vessels with markedly increased peak systolic velocity (PSV; >170 cm/s), end diastolic velocity (EDV; >70 cm/s) and a reduced resistive index (RI = 0.45). Arterialization of the venous waveform was evident along with perivascular random color assignment [Figs. 1 and 2]. The renal artery and vein at the hilum appeared normal. A follow-up after 6 weeks showed spontaneous resolution of the fistula.

## Discussion

The incidence of post-biopsy renal transplant AV fistulas range from 15% to 16%.^1^,^2^ Only a small percentage of these fistulas are sufficiently large in size to warrant surgical intervention for closure.^3^,^4^ Few cases are associated with pseudoaneurysms, which if small in size, resolve simultaneously with the fistula.^2^ The AV fistulas are characterized by a region of high velocity shifts with random color assignment due to vibrating interfaces in the perivascular tissue^5^ and arterialization of venous flow, the latter distinguishing it from focal renal artery stenosis.^1^ Most of the complications occur after renal biopsy, including AV fistulas. The AV fistulas are occluded by transcatheter embolotherapy wherein a steel coil is placed into the fistula from the renal vein approach. This procedure permits nonsurgical closure of the AV shunt without significantly altering the renal function.^6^ Pseudoaneurysms are treated conservatively.^7^ A spontaneous resolution of 75% in AV fistulas was noted within four weeks. All pseudoaneurysms located close to the AV fistulas are also spontaneously closed. In most cases, post-biopsy AV fistulas are clinically occult and resolve spontaneously in 1–2 years.^8^

In most cases, color and duplex Doppler USG easily demonstrate AV fistulas besides other obvious vascular and nonvascular complications and requires invasive procedures such as renal angiography.
